# Response-related sensorimotor rhythms under scopolamine and MK-801 exposures in the touchscreen visual discrimination test in rats

**DOI:** 10.1038/s41598-022-12146-z

**Published:** 2022-05-17

**Authors:** Diána Kostyalik, Kristóf Kelemen, Balázs Lendvai, István Hernádi, Viktor Román, György Lévay

**Affiliations:** 1 Cognitive Pharmacology Laboratory, Department of Pharmacology and Drug Safety, Gedeon Richter Plc., Gyömrői út 19–21, Budapest, 1103 Hungary; 2 Department of Pharmacology and Drug Safety, Gedeon Richter Plc., Budapest, 1103 Hungary; 3 Department of Experimental Zoology and Neurobiology, Faculty of Sciences, University of Pécs, Pécs, 7622 Hungary; 4 Institute of Physiology, Medical School, University of Pécs, Pécs, 7622 Hungary; 5 Grastyán Translational Research Center, University of Pécs, Pécs, 7622 Hungary; 6 Szentágothai Research Center, University of Pécs, Pécs, 7622 Hungary; 7 Department of Morphology and Physiology, Faculty of Health Sciences, Semmelweis University, Budapest, 1085 Hungary

**Keywords:** Problem solving, Pharmacodynamics, Electroencephalography - EEG

## Abstract

The human mu rhythm has been suggested to represent an important function in information processing. Rodent homologue rhythms have been assumed though no study has investigated them from the cognitive aspect yet. As voluntary goal-directed movements induce the desynchronization of mu rhythm, we aimed at exploring whether the response-related brain activity during the touchscreen visual discrimination (VD) task is suitable to detect sensorimotor rhythms and their change under cognitive impairment. Different doses of scopolamine or MK-801 were injected subcutaneously to rats, and epidural electroencephalogram (EEG) was recorded during task performance. Arciform ~ 10 Hz oscillations appeared during visual processing, then two characteristic alpha/beta desynchronization-resynchronization patterns emerged mainly above the sensorimotor areas, serving presumably different motor functions. Beyond causing cognitive impairment, both drugs supressed the touch-related upper alpha (10–15 Hz) reactivity for desynchronization. Reaction time predominantly correlated positively with movement-related alpha and beta power both in normal and impaired conditions. These results support the existence of a mu homologue rodent rhythm whose upper alpha component appeared to be modulated by cholinergic and glutamatergic mechanisms and its power change might indicate a potential EEG correlate of processing speed. The VD task can be utilized for the investigation of sensorimotor rhythms in rats.

## Introduction

Task-related sensory, motor and cognitive processes can evoke short-lasting, regionally localized amplitude reduction (event-related desynchronization, ERD) or enhancement (event-related synchronization, ERS) in the rhythmic activity of alpha (~ 8 to 12 Hz) and beta (~ 15 to 25 Hz) oscillations, displaying specific spatio-temporal localizations depending on task characteristics. Alpha desynchronization is associated to cortical information processing and occurs over task relevant brain areas, whereas task irrelevant regions show synchronization as a proxy for cortical inhibition^1–5^. Alpha-band oscillations are closely linked to sensory information processing and attention, and their causal role have been suggested in the modulation of these processes^4–6^. In addition, alpha rhythm is prone to be changed in people having problems with focused attention^7^, impaired parieto-occipital alpha reactivity is characteristic in neuropsychiatric disorders such as Alzheimer’s Disease (AD)^8^ and schizophrenia^9^.

In humans, somatosensory stimulation^1,10^, and the execution or even the observation of voluntary goal-directed movements such as touch or button pressing can evoke the desynchronization of rhythmic oscillations recorded from the primary sensorimotor cortex^11^. These oscillations, known as the mu rhythm^12^, consist of typically arciform waves, which arise/synchronize during immobile attentive waking limited to brief (0.5–2 s) periods^11^ with dominant frequencies in the alpha band intermixed with beta waves^13^. In human tasks, where subjects have to judge visually presented images and give a motor response, the power of mu rhythm enhances during stimulus presentation and attenuates during the motor response^14^. It is presumed that the mu rhythm largely reflects motor processing, however, recent data suggest that it can also indicate the conjunction of multiple factors associated with sensory and cognitive aspects of motor control^15–18^, thereby having an important function in information processing that connects perception to action^19^. Nevertheless, its proper functional relevance has not been clearly established yet.

It has been demonstrated that the functional analogue of the human mu waves is also present in animals, such as in cats^20^ and monkeys^21^ while in rodents − wherein the face-whisker representation in the somatosensory cortex is particularly large − only limited information is available. Although the existence of mu homolog rodent sensorimotor rhythms has already been suggested in previous studies^22–27^, yet to date no study has detected and investigated them in a complex, training-based cognitive paradigm. The rodent touchscreen Visual Discrimination (VD) test is a discrimination learning task based on operant conditioning^28,29^, wherein animals are trained to choose the correct (rewarding) one of two simultaneously presented visual stimuli by responding with a touch (nose poke) on a visual display. The touch response itself is a voluntary goal-directed action, which terminates in a tactile stimulus and integrates sensory-, movement-, and cognition-related mechanisms with top-down cortical processes^30^. The VD task is frequently used in preclinical cognitive research due to its high translational potential. Since task-related electroencephalography (EEG) in rodents is an excellent tool for the thorough exploration of brain processes and gives the opportunity of pharmacological manipulations, combining the VD task with EEG may reveal the underlying neuronal activity and potential correlates of cognitive functioning^31^.

By the exploration of brain activity underlying correct touch responses in the rat VD task, this study aims to answer the following questions. (1) Can sensorimotor alpha and beta oscillations be detected during the touch responses? (2) How these rhythms change under cognitive dysfunction induced by the manipulation of different neurotransmitter systems? To model transient cognitive impairment, a single dose of the muscarinic receptor antagonist scopolamine, or the NMDA glutamatergic antagonist MK-801 were applied. Cholinergic and glutamatergic neurons abundantly innervate the thalamocortical network, the main route of sensory information processing^32^, and these drugs have already been found to cause profound cognitive dysfunctions in many cognitive paradigms in rodents including the VD^33^. In addition, scopolamine has also been shown to induce cognitive decline administered in healthy young volunteers and has suggested to be a psychopharmacological model of aging and AD^34^. MK-801 has been utilized to model the positive, negative, and cognitive deficits of schizophrenia both in healthy man and laboratory animals^35^. Moreover, these widely used symptomatic models can mimic some disease-specific EEG abnormalities. Namely, scopolamine generally causes a slowing on EEG during resting state, i.e. decreases alpha and beta activity, which leads to enhanced slow frequency oscillations. The similar slowing was also shown in AD patients and believed to be a hallmark of cognitive decline. Decreased functional connectivity between different cortical regions has also been demonstrated in the alpha and beta bands both in AD patients and in humans injected with scopolamine^34,36,37^. In addition, besides the typical gamma enhancing property, MK-801 has been demonstrated to cause an overall increase in the alpha power, which was decreased by atypical antipsychotics on the spontaneous EEG in rats^36,38^.

## Methods

### Subjects

All experimental procedures were approved by the Ethical Committee of Gedeon Richter Plc. and followed the Hungarian Government Decree (40/2013. (II. 14) on the use of animals in research, which is based on the 2010/63/EU Directive on the protection of animals used for scientific purposes. All methods were carried out in accordance with relevant guidelines and regulations and were reported in accordance with the ARRIVE (Animal Research: Reporting of In Vivo Experiments) 2.0 guidelines. Male Lister Hooded rats (n = 12; Envigo, UK) served as subjects in this study. Rats were housed 4 per cage and were kept under controlled environmental conditions (temperature 21 ± 1 °C, humidity 50–60%) and reversed light/dark cycle (lights on from 6 PM to 6 AM). Cages were enriched by wooden chew sticks and paper tunnels. All behavioural measurements were conducted during the active phase. To maintain motivation, mild food restriction was applied to keep the animals’ weight between 85–90% of their free-feeding weight. Rats were ~ 6 to 7-month-old during pharmacological testing.

### Visual discrimination (VD) task

**Figure 1:**
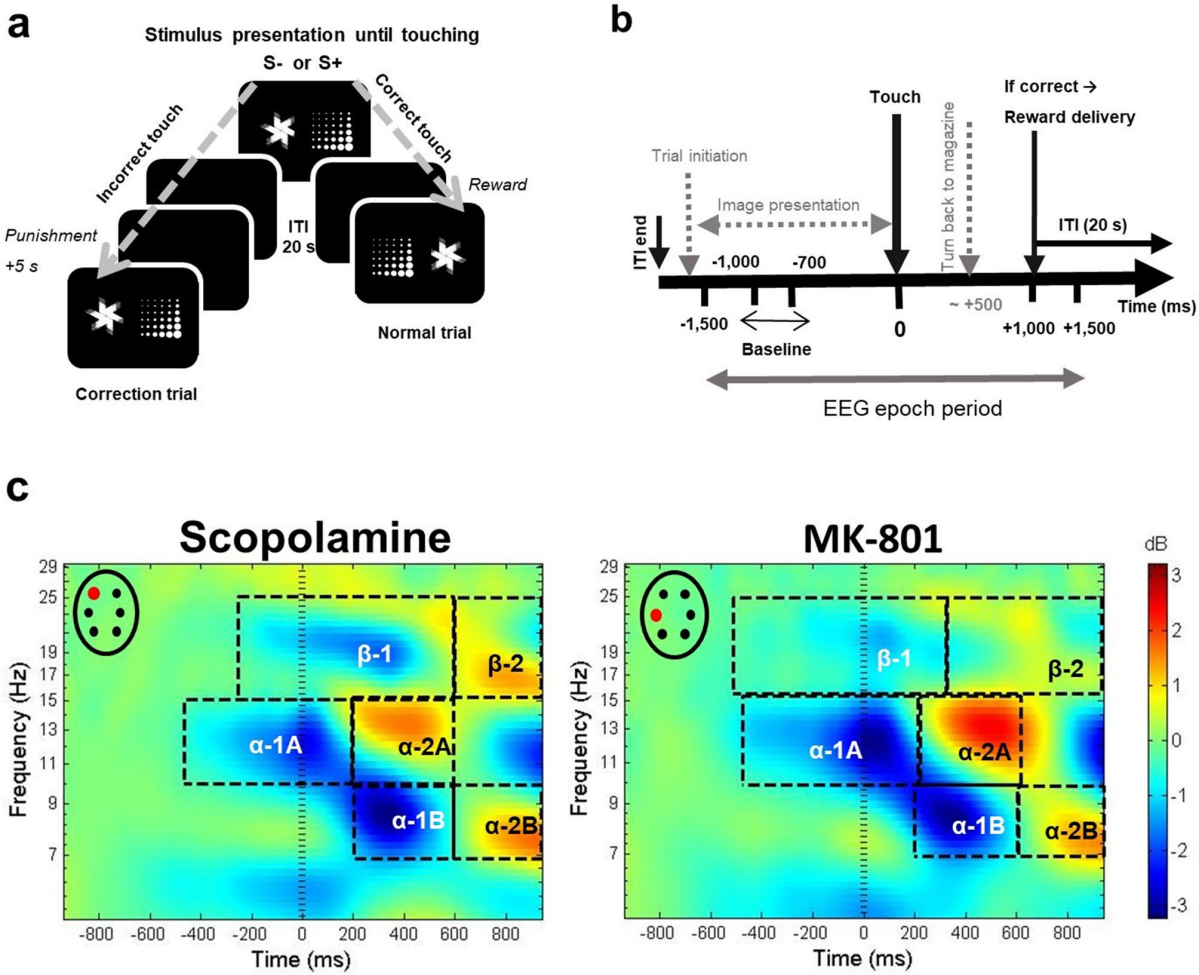
The touchscreen Visual Discrimination (VD) task combined with EEG. (**a**) Graphical illustration of the VD task. Rats discriminated two simultaneously presented visual stimuli on a computer-controlled touch screen. Correct (S+) responses were rewarded by a pellet, and a subsequent intertrial interval (ITI; 20 s) was followed by a normal trial with pseudorandom image positions. Incorrect (S −) responses were punished with a time-out period (5 s), no reward was given, and were followed by correction trials having the same image positions as in the preceding trial until the correct image was touched. Neither correct nor incorrect choices were followed by positive or negative feedback signals. (**b**) The EEG epoch timeline. EEG was time-stamped to touches (t = 0); the 3-s-long (peristimulus +/− 1500 ms) EEG epochs were spectrally decomposed to provide ERSP values. Solid and broken arrows mark events with fix timing and with putative time-course relative to 0, respectively. (**c**) Group average ERSP maps of scopolamine and MK-801 experiments. Plots show the mean time course of ERSP power (decibel, db) change relative to the baseline in the period from ~ 1000 ms before to ~ 1000 ms after the correct touch responses (t = 0) in the frequency range of 5–30 Hz averaged across all subjects and treatment conditions in the scopolamine (n = 9; map on frontal left electrode) and MK-801 experiments (n = 7; map on parietal left electrode) separately. Black rectangles indicate the time–frequency windows of interest. ERSP maps were created by the EEGLab toolbox of MatLab.

Testing was conducted in 12 automated, electromagnetically shielded, touchscreen-equipped operant chambers for rats (Campden Instruments Ltd., Loughborough, U.K.) as described elsewhere^28,29,74^. Rats were pretrained in a stepwise fashion (for the details see^28,74^), then the specific VD task training followed to reach stable performance (72 trials completed with ≥ 80% correct responses on 2 consecutive days). The task (Fig. 1a) required rats to discriminate between two simultaneously displayed visual stimuli (‘marbles’ or ‘fan’) and learn which stimulus is associated with reward. Touching the correct stimulus was rewarded with a food pellet (Dustless Precision Pellets, 45 mg-purified, Bio-Serv, Flemington, US), dropping down opposite to the screen, so rats had to turn around to collect it. Food delivery was delayed by 1 s to avoid artefacts on the EEG and was accompanied by illumination of the tray light. Head entry in the reward tray to collect the reward turned off the light and initiated the intertrial interval (ITI; 20 s). The end of the ITI was indicated by the illumination of the tray light again. The rat had to initiate trials by entering and exit the reward tray, thus the tray light turned off. Touching the incorrect stimulus was punished with a timeout (5 s) and no reward was given. Incorrect responses were followed by correction trials (re-presentation of the same trial) until the rat responded correctly. Correction trials were not counted towards the trial limit or accuracy score. There was neither positive nor negative feedback signal after correct and incorrect touches. Session length was maximum 45 min or the time to complete 72 trials. Correct stimulus positions were determined pseudo-randomly. Reward contingencies of images were counterbalanced between subjects and were kept constant for each rat throughout the whole experiment.

### EEG surgery

After the VD training, animals were equipped with cortical surface electrodes under isoflurane anaesthesia applying a stereotaxic frame. Six tiny stainless-steel screw electrodes (d = 0.8 mm) were implanted epidurally into the skull over the primary motor, somatosensory and visual cortical areas (anterior–posterior and medial–lateral directions in mm referenced to the bregma): frontal left (FL, 2.5 mm, 2 mm) and right (FR, 2.5 mm, − 2 mm), parietal left (PL, − 3, 3) and right (PR, − 3, − 3), occipital left (OL, − 7, 3 mm) and right (OR, − 7, − -3). The reference and ground electrodes were placed over the cerebellum. Electrode wires were soldered to an 8-channel electrode interface board (Multichannel Systems, Reutlingen, Germany), then the whole set was fixed to the skull surface with dental acrylic cement. Animals were allowed 2 weeks to recover from surgery.

### Study design

Twelve rats were trained, operated and participated in the experiments in total. Sample sizes were calculated based on a priori experiments investigating the cognitive effect of scopolamine and MK-801 in the VD test. Previous in-house experiments were utilized for drug titration, so the maximal tolerable dose with certain cognitive disrupting but still acceptable side effects was chosen as the highest. Within-subject study design was applied, where treatment sequences were randomized, so each animal received each treatment in a random order with a minimum 3-day wash-out period (for the detailed treatment regimen see Supplementary Table S1). Drug administrations were conducted in a blinded manner. One month elapsed between the start of the two experiments.

### Experiment 1: Scopolamine

Animals were randomly divided into 4 groups. Scopolamine hydrobromide (Tocris Bioscience) at the dose of 0.025, 0.035 and 0.05 mg/kg (corrected for the salt) dissolved in saline (groups: SCOP 0.025, SCOP 0.035 and SCOP 0.05, respectively) or vehicle (veh) was administered subcutaneously (sc.) in a volume of 1 ml/kg, 20 min before testing.

### Experiment 2: MK-801

Animals were randomly divided into 4 groups. MK-801 maleate (Tocris Bioscience) at the dose of 0.05, 0.0575 and 0.065 mg/kg (corrected for the salt) dissolved in saline (groups: MK 0.05, MK 0.0565, and MK 0.065, respectively) or vehicle (veh) was administered sc. in a volume of 1 ml/kg, 20 min before testing.

### EEG data acquisition and pre-processing

Electrophysiological signals were wirelessly recorded from six active electrodes by small-size headstages (W8-HS-SR) connected to 100 mAh batteries provided by Multichannel Systems (Basic Wireless System, Harvard Bioscience, Inc., USA). Signal amplification (bandwidth: 1 Hz to 5 kHz) and digitalization (sampling rate 10 kHz per channel with 16-bit precision) were processed right on the head, and digital signals were transferred via radio communication (2.4 GHz). Multichannel Experimenter software (2.1.4) was used for on-line data acquisition and recording. Behavioural, electrophysiological, and video data were synchronized and exported for analysis applying the Multichannel Analyzer and DataManager (2.1.4) software. For off-line data pre-processing we used the MatLab programming environment (R2013b, The MathWorks, Inc., Natick, MA USA) with built-in and self-developed scripts as well as the EEGLab (13.4.4b) toolbox^75^. Raw data were band-pass filtered with cut-offs of 1 Hz and 80 Hz, and notch filter with passband edges at 45–55 Hz was applied. After filtering, data were manually scanned for contamination by muscular and electrode artefacts then resampled to 1 kHz. Broad epochs were defined ranging between − 1500 and + 1500 ms relative to the correct touches (t = 0), presuming that cognitive processing underlying perception, discrimination, decision, and execution occurred during this period (for the EEG epoch timeline see Fig. 1b). Independent component analysis (ICA) method^76^ was used to select systematic artefacts. Finally, data were re-referenced to the average reference. To estimate response-related shifts in the power spectrum, EEG epochs were decomposed into their time–frequency representation by EEGLab using a set of Morlet wavelets (3 0.8), generating 400 time points (-942 ms to + 941.5 ms) and estimating 100 log-spaced frequencies from 3.0 Hz to 80.0 Hz. The estimated frequency-band-specific power at each time point was decibel normalized using the pre-response interval from − 1000 to − 700 ms as baseline. The baseline period was chosen because it was free of the various unpredictable movements that can occur during ITI, since rats stood still in front of the screen and looked at the images. Furthermore, in terms of cognitive processing this period can be considered homogeneous. Single trial ERSP values were also calculated using the same parameters, which was necessary for establishing relationship between ERSP and reaction time (correct response latency), a trial specific variable. The present study focused on the rhythmic activity in the alpha (7–15 Hz) and beta (16–30 Hz) bands.

### Data analysis

Twelve rats were used in the experiments in total, but due to broken electrodes, disrupted cognitive performance or inappropriate EEG signals some rats had to be excluded from the analyses. So finally, 9 animals were used in the behavioural and 8 in the EEG analysis of scopolamine, and 7 rats were analysed in the MK-801 experiment.

### Behaviour

Data acquisition was made by WhiskerServer (Cambridge University Technical Services Ltd., U.K.) and Abet II VideoTouch (Campden Instruments Ltd., Loughborough, U.K.) software. For both compounds three doses were compared to vehicle. The main cognitive measures were (1) Correct %: percent of correct choices; (2) Correct latency (reaction time): average time required to touch the correct image following presentation of stimuli. Surrogate measures were as follows: (3) Incorrect latency: average time required to touch the incorrect image following presentation of stimuli (4) Reward latency: average time required to collect the reward. (5) All trial: the number of all trials including corrections. (6) Correction trials: the number of all correction trials. (7) ITI touches: the number of touches during the ITI.

Behavioural data were evaluated by repeated measures one-way ANOVA (repeated: subjects) followed by Dunnett’s post hoc tests or by non-parametric Friedman test followed by Dunn’s multiple comparisons if homogeneity of variances was violated. All treatment groups were compared to the appropriate control group, and p values < 0.05 were defined as statistically significant. Statistical analysis and graphs were performed using GraphPad (Prism, version 8, GraphPad Software, Inc., USA) software.

#### EEG

Since our testing protocol required rats having stable high baseline cognitive performance normally (≥ 80% correct), relatively few incorrect responses were detected and large trial number differences occurred (Supplementary Table S2 summarizes trial counts), As these factors could bias the ERSP results, incorrect touch responses were not included in this study. Condition average ERSP maps of correct touches in the scopolamine and MK-801 experiments were separately analysed. The relevant time–frequency (TF) windows were selected by visual inspection (see Fig. 1c for the group-average ERSP plots at the FL and PL electrode sites in the scopolamine and MK-801 experiment, respectively. Supplementary Figure S8 and S10 show all ERSP maps). The most powerful alterations were found in the alpha band (7–15 Hz), oscillations formed a spatiotemporal pattern displaying characteristic spectral topography of the response-related activity in both experiments. Therefore, the same alpha TF windows, termed as cycles, were chosen and statistically evaluated throughout the whole study: alpha-1A (ERD, 10–15 Hz, from − 450 to + 200 ms), alpha-1B (ERD, 7–10 Hz, from + 200 to + 600 ms), alpha-2A (ERS, 10–15 Hz, from + 201 to + 600 ms) and alpha-2B (ERS, 7–10 Hz, from + 601 to + 942 ms). Power changes in the beta range were less consistent, TF windows slightly varied with treatment conditions, so were defined individually in each experiment: experiment 1: β-1: ERD, 16–25 Hz, from − 350 to + 600 ms; β-2: ERS, 15–20 Hz, from + 600 to + 942 ms; experiment 2: β-1: ERD, 15–25 Hz, from − 500 to + 350 ms, β-2: ERS, 15–25 Hz, from + 350 to + 942 ms). ERSP data were analysed by repeated measures two-way ANOVA (factors: treatment, channel (repeated) and subject (repeated)) with Greenhouse–Geisser correction and was followed by Dunnett’s multiple post hoc comparisons.

To explore the relationship between reaction time and ERSP, we performed a linear mixed effects analysis for each of the two experiments. Linear mixed models (LME) are an extension of linear models adding random terms. Through random effects we can take into account that measurements for the same subject are not independent. As fixed effects, we entered ERSP change, treatment, and channels into the model with all interactions. As random effect, we had intercepts for subjects. The effect of power change was assessed using likelihood ratio tests of the full model against the model without ERSP change. This test compares the likelihood of two alternative models. Its significance means that adding power change to the explanatory variables makes the predictability of reaction time better as compared to the model without power change. Trials with artefacts in the EEG signal around the touch event were not included in the data. Due to atypical response execution and because vertical outliers can have a disproportionate effect on regression lines, touches required ≥ 5 s were also excluded from this analysis. Visual inspection of residual plots did not reveal any obvious deviations from homoscedasticity or normality.

A separate analysis was performed in control animals summarizing all data from both experiments (n = 16). The aim of this drug-free analysis was to establish the morphology, spectral characteristics, spatial distribution, and functional reactivity of the touch response-related oscillations. ERSP was calculated for alpha (see above) and beta (β-1: 16–25 Hz, from − 200 to + 550 ms; β-2: 16–19 Hz, from + 720 to + 942 ms), TF windows selected by visual inspection.

The epoch-wide FFT spectra (Hamming window, frequency resolution 0.97 Hz) and the maximal power of alpha frequencies (at the peak) were evaluated at each electrode site, which were compared with one-way ANOVA followed by Tukey post hoc comparison tests.

Statistical analysis and graphs were performed using R (R Core Team, 2018) and lme4 (Douglas Bates, Martin Maechler, Ben Bolker, Steve Walker, 2015) and GraphPad (Prism, version 8, GraphPad Software, Inc., USA) software. ERSP figures were created by the EEGLab (13.4.4b) toolbox^75^ of MatLab programming environment (R2013b, The MathWorks, Inc., Natick, MA USA).

## Results

### Drug-free analysis

**Figure 2:**
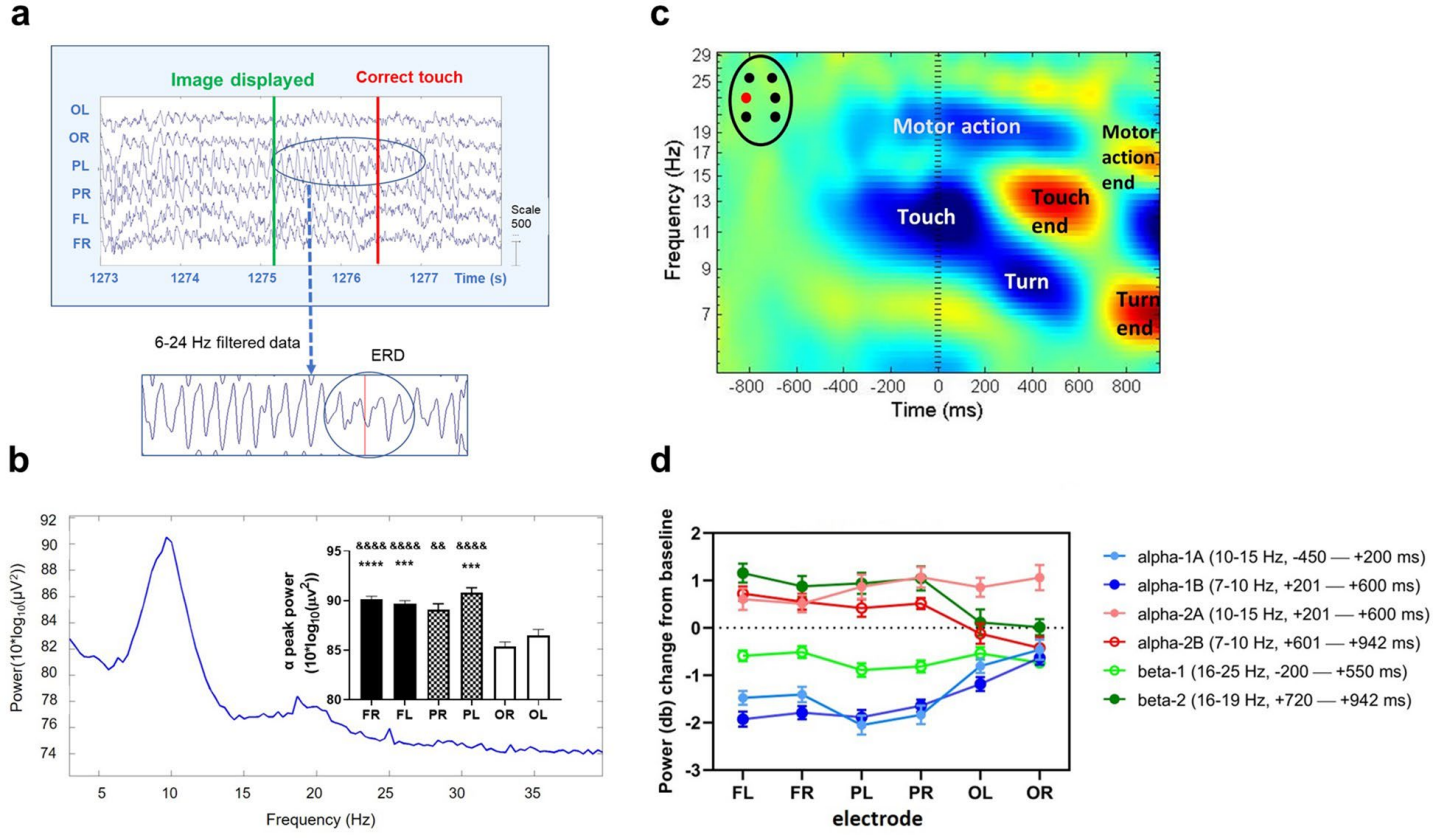
Drug-free analysis: alpha and beta oscillatory dynamics in the normal brain. (**a**) Raw EEG data around a typical correct epoch. Image presentation was followed by a burst of arciform alpha configuration predominating over the parietal left (PL) electrode. Arciform waveforms emerged in the alpha range during image presentation, then alpha desynchronization appeared right before and over the course of the touch. The 6–24 Hz filtered segment more specifically illustrates the region of interest. (**b**) FFT spectra. The plot represents a dominant peak in the alpha (9–10 Hz) and a smaller amplitude beta peak (18–20 Hz) of correct epochs at the PL electrode. The graph shows the frontoparietal dominance of alpha power at the peak (mean ± SEM) over each electrode. && p < 0.01 and &&&& p < 0.0001: significant multiple comparisons tests compared to OR channel; *** p < 0.001 and **** p < 0.0001: significant multiple comparisons tests compared to OL channel. (**c**) ERSP map of correct touches at the PL electrode. Plots show the mean time course of ERSP power (decibel, db) change relative to the baseline in the period from ~ 1000 ms before to ~ 1000 ms after correct touch responses (t = 0) in the frequency range of 5–30 Hz averaged across all subjects (n = 16) treated with vehicle; motor functions associated to the ERD and ERS cycles were denoted. The ERSP map were created by the EEGLab toolbox of MatLab. (**d**) Spatial distribution of ERSP power during correct responding. Graph show ERSP change from baseline in all relevant time–frequency windows (db, mean ± SEM; represented by coloured lines) at each electrode in correct epochs. The magnitude of power changes showed frontoparietal dominance in all cycles except for alpha-2A and beta-1, which spread evenly across the cortex. Abbreviations: *FL* frontal left, *FR* frontal right, *PL* parietal left, *PR* parietal right, *OL* occipital left, *OR* occipital right electrodes.

During image presentation biphasic arc-shaped waveforms emerged in the alpha range (9–10 Hz) mainly over the parietal and frontal electrodes synchronously. The wave amplitude (~ 300–500 µV) markedly decreased just before and during the touch (Fig. 2a represents typical EEG data recorded in a correct trial). The FFT spectra of correct epochs (Fig. 2b) revealed a dominant peak in the alpha (9–10 Hz) and a weaker one at twice this frequency in the beta band (18–20 Hz). The peak power of alpha was significantly higher over the frontoparietal sites than over the occipital ones (F_3.031,42.43_ = 21.27, p < 0.0001, see Fig. 2b for significant post hoc results).

Figure 2c shows the ERSP map of correct responses at the PL site. During the correct touches, the EEG displayed desynchronized EEG activity (ERD) surrounding the moment of touch (rearing up and nose-poking to the screen) in the upper range of alpha (10–15 Hz, alpha-1A), which turned into a synchronization (ERS, alpha-2A). The same phenomenon was detected immediately after the first ERD-ERS pattern in low alpha (7–10 Hz, alpha-1B and alpha-2B), which coincided with a complex motor action, coming down onto four limbs and turning around to collect the reward. Alpha cycles coexisted with similar beta dynamics, an ERD developed in β-1 followed by an ERS in β-2. ERSP reactivity showed spatial-dependence during correct responding, as the magnitude of power changes showed frontoparietal dominance in all TF cycles, excluding the alpha-2A, which distributed rather uniformly across the cortex (Fig. 2d).

### Experiment 1: Effects of scopolamine

#### Behaviour

**Figure 3:**
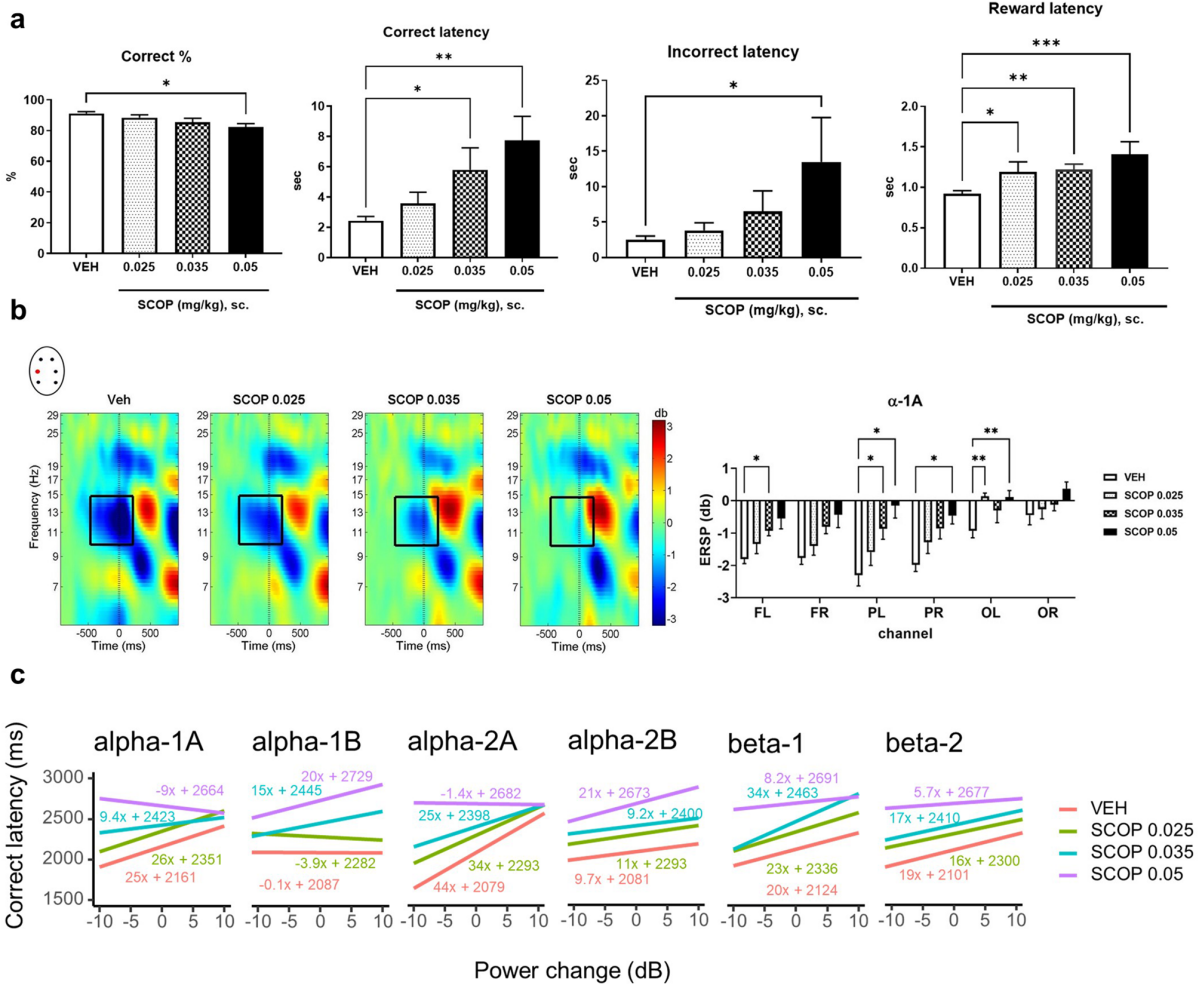
Experiment 1: Effects of scopolamine. (**a**) Behaviour. Scopolamine slightly decreased the percent of correct choices and elongated response latency times mainly at the higher doses, and enhanced reward collection latency at all doses applied. Data represent mean ± SEM values. * p < 0.05, ** p < 0.01 and *** p < 0.001 show significant multiple comparisons test effects of groups treated with different doses of scopolamine compared to the vehicle group. (**b**) ERSP power. ERSP plots represent the power change from baseline (decibel, db) in each group separately at the PL electrode; black rectangles highlight alpha-1A cycle (10–15 Hz, − 450 – + 200). The graph shows the effect of scopolamine in the alpha-1A cycle at each electrode, separately. Note that alpha-1A ERD is very robust in vehicle condition, which gradually decreases with increasing scopolamine dose. Data represent mean ± SEM values, asterisks indicate significant differences compared to the control: *p < 0.05 and **p < 0.01. Abbreviations: *FL* frontal left, *FR* frontal right, *PL* parietal left, *PR* parietal right, *OL* occipital left, *OR* occipital right electrodes. ERSP maps were created by the EEGLab toolbox of MatLab. (**c**) Single-trial ERSP and reaction time relationship. Plots show the dependence of reaction time (correct latency) on ERSP power in each time–frequency domain after vehicle or different doses of scopolamine based on the linear mixed effect analysis (LME). The regression lines are focal predictor effects from a fitted LME model with treatment and channel as fixed effects and subject as random effect. Slopes of regression lines show effect size (the predicted change in correct latency for unit ERSP power change), while y-intercept values are used to show how treatment modulates correct latency in general.

Scopolamine treatment caused a dose-dependent cognitive impairment via decreasing the percent of correct responses and increasing correct latency (reaction time) (Fig. 3a; F_3,24_ = 3.28, p < 0.05 and F*r* = 11.53, p < 0.01, respectively). In addition, incorrect responses also slowed down in a dose-dependent manner (Fig. 3a, F*r* = 9.13, p < 0.05). The 0.025 mg/kg dose did not significantly affect the cognitive measures, while the 0.035 mg/kg dose accelerated merely correct responding and the highest 0.05 mg/kg dose had robust effects on both correct % and correct and incorrect latency times compared to vehicle. A slight decrease in motivation and/or in motor activity can be suggested according to the deceleration in reward collection (Fig. 3a, F*r* = 18.33, p < 0.001). Supplementary Fig. S3 shows graphs of surrogate measures (all trials, correction trial number, ITI touch number), which did not change significantly after scopolamine administration indicating that neither a sharp decrease in activity level nor an increase in repetitive touch behaviour was present. See Supplementary Fig. S4 for data distribution plots of each behavioural parameter.

#### ERSP

Scopolamine increased the power in the alpha1-A ERD cycle at the time of the correct touch responses. The desynchronization, emerging predominantly over the frontoparietal regions in the control group, was dose-dependently suppressed by scopolamine, showing significant treatment and channel effects (Fig. 3b; F_1.427, 9.988_ = 6.60, p < 0.05 and F_2.736, 19.15_ = 14.28, p < 0.0001, respectively) with no interaction. Frequency shifts in oscillatory activity in the other cycles remained the same as in the control group or showed only non-significant enhancement by scopolamine. The entire list of ERSP data across the relevant time–frequency windows for each electrode and drug dose can be found as Supplementary Table S7 online.

#### Relationship between single-trial ERSP and reaction time (correct latency)

Regression analysis showed that alpha and beta power change were strongly connected to correct touch latency times. Based on likelihood ratio tests, ERSP change explained a significant proportion of variance in reaction time (p < 0.0001 in each TF window, see Supplementary Table S11 for likelihood-ratio test results). Regression line slopes indicated that the larger the power increment was in vehicle-treated animals, the slower the animals responded to the visual stimuli, especially in the first alpha ERD-ERS pattern (alpha-1A, alpha-2A). Slighter changes were observed in the beta (beta-1, beta-2) and in the second alpha ERD-ERS cycle (alpha-1B, alpha-2B; Fig. 3c). In the case of scopolamine, the slopes generally pointed in a positive direction in all TF windows, however, the highest dose of scopolamine caused slight inverse correlations in alpha-1A and alpha-2A cycles. The effect sizes of different scopolamine doses were diverse and did not increase proportionally with increasing dose; the largest effects appeared mostly in alpha-2A and beta-1 cycles (Fig. 3c).

### Experiment 2: Effects of MK-801

#### Behaviour

**Figure 4:**
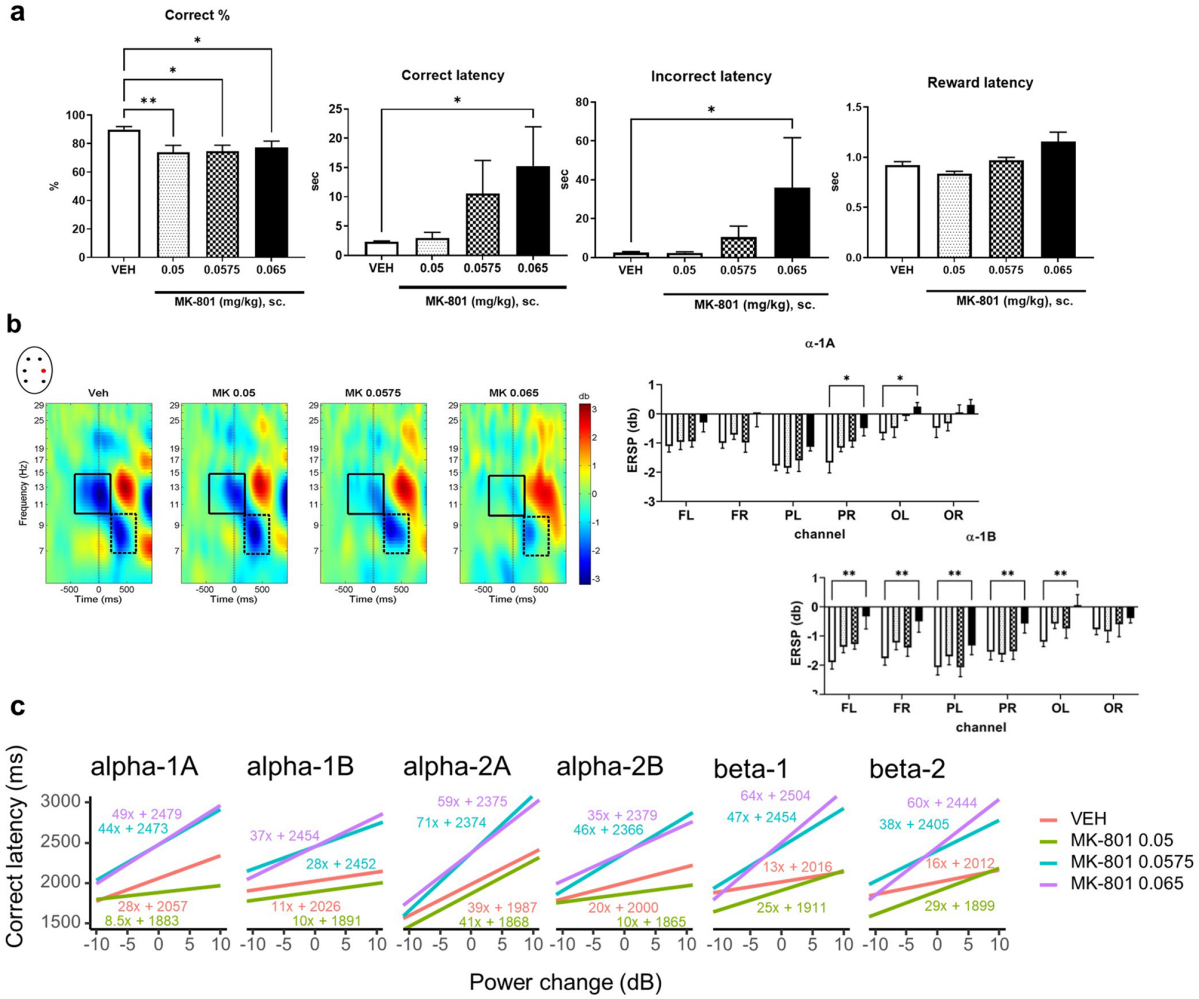
Experiment 2: Effects of MK-801. (**a**) Behaviour. MK-801 decreased the percent of correct choices and elongated the response latency times mainly at the highest dose without affecting reward collection latency. Data represent mean ± SEM values. * p < 0.05 and ** p < 0.01 show significant multiple comparisons test effects of groups treated with different doses of MK-801 compared to the vehicle group. (**b**) ERSP power. ERSP plots represent the power change from baseline (decibel, db) in each group separately at the PR electrode; black rectangles highlight the alpha-1A (10–15 Hz, − 450 – + 200). and alpha-1B (7–10 Hz, + 200–600) cycles. Graphs show the effect of MK-801 in the alpha-1A and alpha-1B cycles at each electrode, separately. Data represent mean ± SEM values, asterisks indicate significant differences compared to the control: *p < 0.05 and **p < 0.01. Note that both alpha-1A and alpha-1B ERD are very robust in vehicle condition, which gradually decline with increasing scopolamine dose. Abbreviations: *FL* frontal left, *FR* frontal right, *PL* parietal left, *PR* parietal right, *OL* occipital left, *OR* occipital right electrodes. ERSP maps were created by the EEGLab toolbox of MatLab. (**c**) Single-trial ERSP and reaction time relationship. Plots show the dependence of correct latency (reaction time) on ERSP power in each time–frequency domain after vehicle or MK-801 treatment based on the linear mixed effect analysis (LME). The regression lines are focal predictor effects from a fitted LME model with treatment and channel as fixed effects and subject as random effect. Slopes of regression lines show effect size (the predicted change in correct latency for unit ERSP power change), while y-intercept values are used to show how treatment modulates correct latency in general.

MK-801 induced cognitive deficit by the reduction of the percent of correct choices at all doses applied and by the elongation of correct and incorrect latency times, wherein only the highest dose was significant (Fig. 4a; F*r* = 9.17p < 0.05, F*r* = 15.34, p < 0.01 and F*r* = 9.69, p < 0.05, respectively). Although treatment effect was significant (F*r* = 13.11, p < 0.01), post hoc comparisons did not show any significant differences in reward collection times (Fig. 4a), therefore motivational and/or motor disturbance were not suggested. Increment in correction trial numbers (F*r* = 9.78, p < 0.05), especially in the MK 0.05 group, might be the consequence of both larger incorrect trial number and cognitive rigidity. In addition, neither the number of all trials nor ITI touch number declined after any MK-801 doses. For graphs of surrogate measures see Supplementary Fig. S5. Supplementary Fig. S6 shows data distribution plots of each behavioural parameter.

#### ERSP

MK-801 enhanced the power in both alpha-1A and alpha-1B ERD cycles, particularly at the highest dose (Fig. 4b). Significant treatment (alpha-1A: F_2.682, 16.09_ = 6.86 p < 0.01; alpha-1B: F_2.158, 12.95_ = 8.29, p < 0.01) and channel effects (alpha-1A: F_3.305, 19.83_ = 14.80, p < 0.0001; alpha-1B: F_2.635, 15.92_ = 8.52, p < 0.01) were shown with no interaction. None of the other alpha and beta cycles were significantly affected by MK-801, frequency shifts in oscillatory activity remained the same as in the control group or showed only non-significant trend of enhancement. The entire list of all the ERSP data across the relevant time–frequency windows for each electrode and drug dose can be found as Supplementary Table S9 online.

#### Relationship between single-trial ERSP and reaction time (correct latency)

Regression analysis showed that alpha and beta power change were strongly connected to correct touch latency times. Based on likelihood-ratio tests, ERSP change explained a significant proportion of variance in correct latency time (p < 0.0001 in each TF window, see Supplementary Table S11 for likelihood-ratio test results). The larger the power increment was in vehicle-treated animals, the slower the animals responded to the visual stimuli especially in the first alpha cycle (alpha-1A, alpha-2A), and slighter increases were observed in beta (beta-1, beta-2) and in the second alpha ERD-ERS pattern (alpha-1B, alpha-2B). In the case of MK-801, the slopes generally pointed sharply in a positive direction in all TF windows; the largest effects appeared mostly in alpha-2A. Effect sizes increased proportionally only in beta cycles (Fig. 4c).

## Discussion

Rodent models are indispensable to understand the regulation and functional significance of brain rhythms. In the present study we investigated the correct touch response-related alpha and beta oscillations in rats during the VD task. This approach delivered key findings as follows: i) touch-related sensorimotor alpha and beta oscillations shared similar features with the human mu rhythm; ii) in parallel with cognitive impairment, both scopolamine and MK-801 caused a marked suppression in the upper alpha reactivity for desynchronization; iii) responding speed was strongly associated with sensorimotor alpha/beta power change; generally, the larger the power increment was the slower rats reacted to the correct stimuli.

In the present study, we detected arciform ~ 10 Hz oscillations mainly over the sensorimotor cortical regions during image presentation in a motionless state. Visual regions showed lower alpha amplitude likely due to attenuated gating mechanisms for visual processing underlying image perception and discrimination as a primary process required for task execution. This is in line with the functional concept of the mu rhythm, which emerges during attentive immobile waking and is presumed to reflect sensorimotor information processing and not directly engaged in visual processing when sensorimotor networks become “idle” and synchronized^3,39^.

Correct responses induced the desynchronization of the upper (10–15 Hz) and lower alpha (7–10 Hz) frequencies, which can be functionally connected to touch and turn, respectively. Our results are consistent with findings suggesting the existence of a mu rhythm analogue sensorimotor rodent rhythm. Marini et al.^24^ detected arc-shaped 7–12 Hz oscillations in alert immobile rats, which were blocked before and during a movement. Furthermore, tactile whisker stimulation induced the desynchronization of local field potentials in the 5–12 Hz^26^ or 7–12 Hz^25^ frequency bands recorded over the barrel cortex, or in the 7–14 Hz range detected epidurally over the somatosensory areas^27^. In addition, spontaneous arc-shaped 7–12 Hz oscillations occurred in alert immobile rats and at the transition from rapid eye movement sleep to waking, which were blocked during movement^24^. The 7–12 Hz rhythm was also described at single-unit level in the trigeminal system and was associated with tactile exploratory movements and sensory information processing^23^. Contrary to previous studies, here we observed two movement-related ERDs of alpha activity serving apparently distinct motor functions. In line with our results, two different mu rhythm subtypes in the alpha band were demonstrated in humans, a somatotopically more specific one found in the upper (10–12 Hz) and a widespread, somatotopically non-specific subtype in the lower (8–10 Hz) alpha range. It was speculated that the upper alpha component might reflect a mechanism responsible for selective attention to a specific motor subsystem, whereas the lower component is rather for general processes, activated by different motor behaviour but not necessarily critical to support a specific movement^40^.

As alpha synchronization is presumed to reflect cortical inactivation and the termination of a motor act^3^, we propose that the touch-related upper and lower alpha ERS cycles reflect the cease of the action of touch and turn, respectively. Interestingly, the alpha-2A ERS showed a topographically distinct pattern from other cycles as emerged to the same extent across all electrodes including the occipital sites. One possible reason for that may be that stimuli displayed on the screen from trial start suddenly disappeared after the touch, which resulted in visual brain areas switching into an “idling” state. But this is not the only explanation considering that visual alpha can also be supressed without visual stimulation in a completely dark room in response to eye-opening^41^. Since alpha power has been assumed as an electrophysiological proxy for attention allocation^42,43^, we can speculate that this widespread inhibition might indicate the allocation of attentional locus from one discrete motion to another including top-down control processes.

In this study, besides the dominant alpha (~ 10 Hz), a weaker beta (~ 20 Hz) peak was also shown on the quantitative spectra, which is in alignment with studies verifying that the mu rhythm does not consist of alpha waves only. It is rather composed of alpha and beta frequencies with two characteristic spectral peaks around 10 and 20 Hz, respectively^13,44^. While the alpha component was suggested to have a predominant sensorimotor function, the beta was more closely associated with motor control^19,45,46^. These frequency domains have functionally related yet distinct responsivity patterns. Their activity is often highly correlated especially in movement-related studies^47,48^, however, they do not change at the same rate and there can be slight differences in timing^45,49^. Supporting these findings, we observed that alpha and beta power changed in a closely coupled manner, albeit a single beta ERD-ERS cluster covered the two-fold alpha pattern with slight alterations in timing. In line with our results, desynchronization of somatosensory beta rhythm was previously shown following tactile stimulation in both humans^27,50^ and rats^27^, however, rebound resynchronization, which is typical of humans, has not been detected in rodents yet. As mu rhythm reactivity was shown to be sensitive to task complexity and expertise^51–54^, we suppose that training-based paradigms like the VD might reveal sensorimotor power changes in a greater magnitude than those related to tactile stimulation only.

Evidence suggests that oscillatory alpha rhythm is prone to be changed in people having problems with focused attention^7^. Impaired visual (parieto-occipital) alpha reactivity-poor power suppression to eye-opening—is characteristic of AD^8^ and schizophrenia^9^, and decreased alpha amplitude was associated with cognitive health in the aged population^55^. In humans, mu rhythm reactivity was interpreted as analogous to that of upper alpha in memory tasks, in which desynchronization during retrieval was particularly large over parietal areas, which might be closely related to the storage of semantic and episodic memory traces^56^.

From the cognitive point of view, touch-responding in the VD task is a skilled context-dependent motor trace that has to be retrieved from the reference memory together with the rule of the task itself, namely that food reward can be earned by selecting the correct image. For appropriate response selection, in addition to the successful encoding and discrimination of visual information, cognitive control mechanisms like adjusting the level of attention and applying the correct motor program are required. Based on our results, concurrently with cognitive impairment, both scopolamine and MK-801 suppressed the reactivity of the touch-related upper alpha ERD, which supports the influence of the muscarinic and glutamatergic systems in the regulation of alpha oscillations (see for review:^57^). It is thought that alpha rhythm is generated through cortical interactions with or without the need for thalamic input^58^ and may stem from rhythmic GABAergic interneurons, which may themselves receive input from excitatory output neurons^59^. Cholinergic and glutamatergic neurons abundantly innervate the thalamocortical network, the main route of sensory information processing^32^. In line with our results, increased alpha power was found after a single dose of scopolamine in freely-moving rats^60^, however, others reported no significant effect on this frequency range^61^. Furthermore, MK-801 enhanced the alpha network activity in rats, which was associated with increased activity level^62^. In contrast, in humans both muscarinic and NMDA blockers typically reduce the parieto-occipital alpha oscillations although during the resting state condition^57^. These findings let us to speculate that alpha modulation via these neurotransmitter systems might depend on the activity level and/or the top-down drive required for the execution of a specific task. Considering that alpha desynchronization is essential for accurate task performance and its size is assumed to reflect the magnitude of cortical activation^53^, the reduced alpha response might be indicative of impaired cortical activation thereby the loss of attentional control and/or disturbed sensory information processing. This is in accordance with enhanced sensory-gating mechanisms found in rodents after both MK-801^63^ and scopolamine^64^.

While the upper range of parieto-occipital alpha is considered to relate closely to active cognitive processing, the topographically more widespread lower alpha domain is associated with general attentional demands^65^. Differential regulation of the lower and upper alpha domains was visible in our hands as MK-801 but not scopolamine reduced the lower alpha ERD, which further supports the existence of two mu-alpha rhythm subtypes^40^ in rats and raise the possibility of their distinct neuronal regulation. Contrarily to alpha, neither scopolamine nor MK-801 affected markedly the beta oscillations suggesting different control mechanisms. Although beta oscillations are presumed to be the part of the mu rhythm, the source for mu-alpha and mu-beta seems to be different^66^. Furthermore, as their power completely dissociated in previous cognitive studies (speech perception), a functional distinction has also been suggested^48,67^.

Reaction time is an important factor in relation to the integrity and efficiency of brain functions such as those involved in attention, cognition, and perception. Processing speed tends to slow with age^68^ and cognitive decline in a wide range of brain disorders such as in AD^69^ and schizophrenia^70^. According to our results, connection between reaction time and touch-related alpha/beta power can be presumed, since the LME analysis demonstrated that slower responding was associated predominantly with ERSP power increment both in vehicle condition and under drug exposure. However, the highest scopolamine dose caused slight inverse correlations in the first alpha ERD-ERS pattern, which may be due to a higher level of non-specific motor and motivational decline in this group. In line with our results, Leventhal et al.^71^ demonstrated that increased beta oscillations originating from the basal ganglia well correlated with changes in reaction time during a cued movement paradigm in rats, which is consistent with the fact that in humans increased beta power is associated with slower movements^72,73^. Furthermore, a positive correlation was observed between prestimulus alpha power and reaction time in macaques in an auditory oddball task over the inferotemporal cortex, while a negative association was obtained in the visual cortex^6^. Many earlier studies revealed though that reaction time rather depends on the phase of alpha waves during stimulation^65^.

In conclusion, we suppose that the alpha/beta spectral dynamics encompassing correct touch responses in the VD test certainly reflects the electrophysiological hallmarks of a complex motor action, as the consequence of high-level mental effort and are akin to the human mu rhythm with regards to morphology, spectral contents, spatial distribution, and reactivity. Furthermore, cycles of alpha and beta desynchronization and synchronization can represent the dynamic interplay of turning “off” and “on” neuronal assemblies responsible for accurate task execution. Cognitive dysfunction was accompanied with suppressed upper alpha reactivity, whose generation can be related to both muscarinic and glutamatergic mechanisms. As cognitive slowing related to alpha/beta power increment, sensorimotor rhythms might be potential EEG correlates of processing speed. It remains to be verified in the future whether clinically proven cognitive enhancer drugs can reverse the electrophysiological effects found in these dementia models. In addition, it must be noted that due to the main limitation of this study, namely that reliable analysis of incorrect trials was not possible, future research is needed using suitable experimental design (e.g. continuous EEG recording in the course of the VD learning period) to clarify the relationship between alpha reactivity and choice behaviour.

## Supplementary Information

Supplementary Information.

## Data Availability

The datasets generated and analysed during the current study are available in the G-Node data repository.
