# Supplementary material for: Response-related sensorimotor rhythms under scopolamine and MK-801 exposures in the touchscreen visual discrimination test in rats
Source: Sci Rep. 2022 May 17;12:8168. doi: 10.1038/s41598-022-12146-z (PMC9114334; doi:10.1038/s41598-022-12146-z)
Supplement: Supplementary file 1 — Supplementary Information. [file 41598_2022_12146_MOESM1_ESM.docx]

# Response-related sensorimotor rhythms under scopolamine and MK-801 exposures in the touchscreen Visual Discrimination test in rats

Diána Kostyalik, Kristóf Kelemen, Balázs Lendvai, István Hernádi, Viktor Román and György Lévay

**Supplementary Table S1.** The table represents the applied treatment sequence in the within-subject experimental design in both experiment 1 and 2. One month elapsed between the experiments.

**Supplementary Table S2.** The table shows the sum of all trials used in the behavioural and EEG analyses. Note that there is a large difference between correct and incorrect conditions, therefore ERSP and regression analyses were limited on correct trials.

**
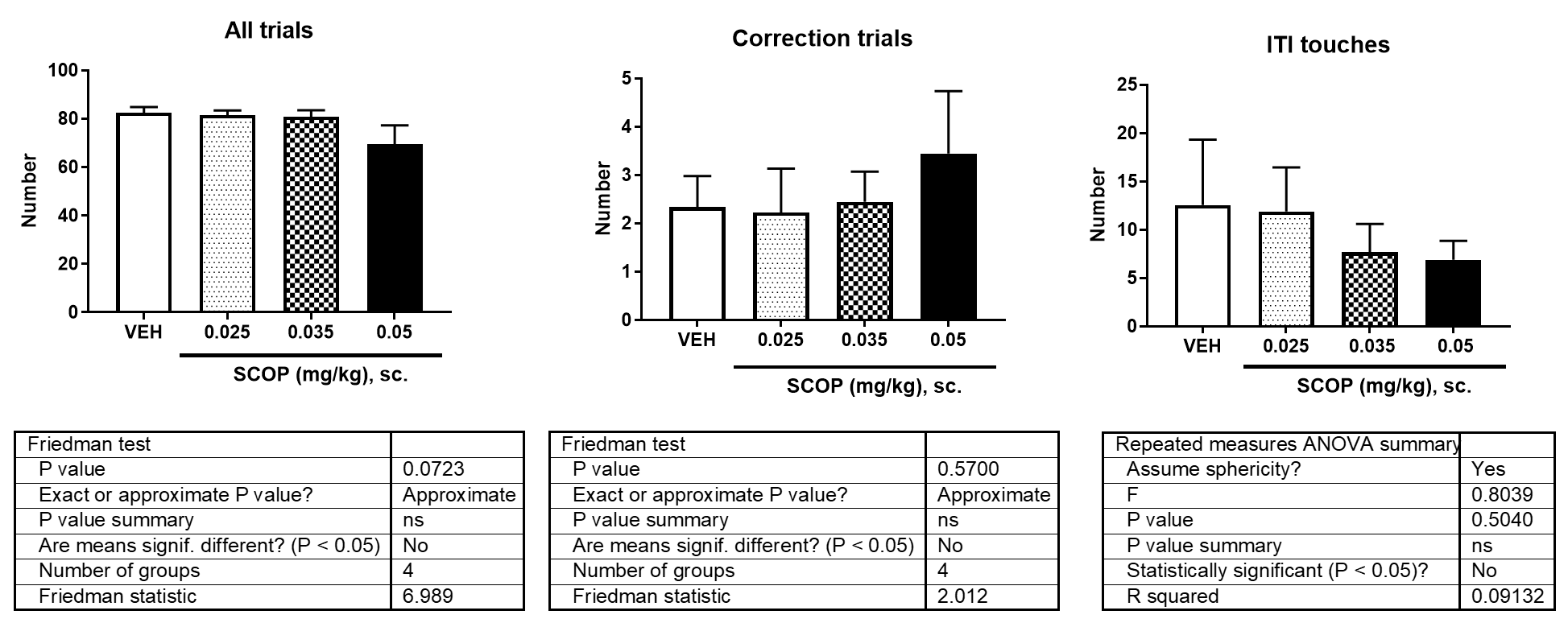
**

**Supplementary Figure S3. Surrogate measures of the scopolamine experiment.** Data represent mean ± SEM values (n=9). All trial: the number of all trials including corrections**;** Correction trials: the number of all correction trials; ITI touches: the number of all touches to the screen (either on the left or right response window) during the intertrial interval (ITI).


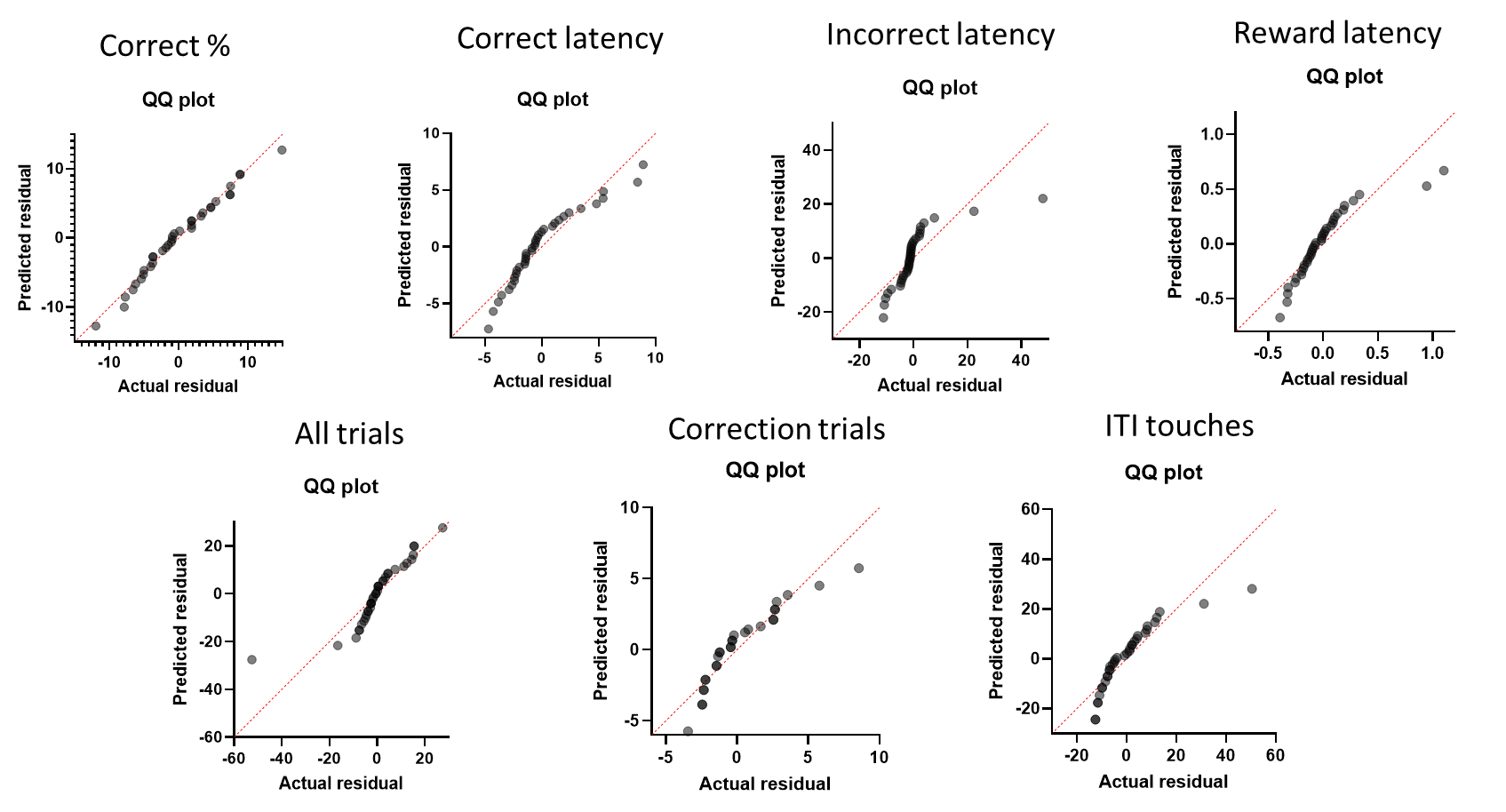


**Supplementary Figure S4. Data distribution of behavioural parameters in the scopolamine experiment.** Quantile-Quantile (QQ) plots show data deviations from normal distribution in all parameters except for Correct%.

**
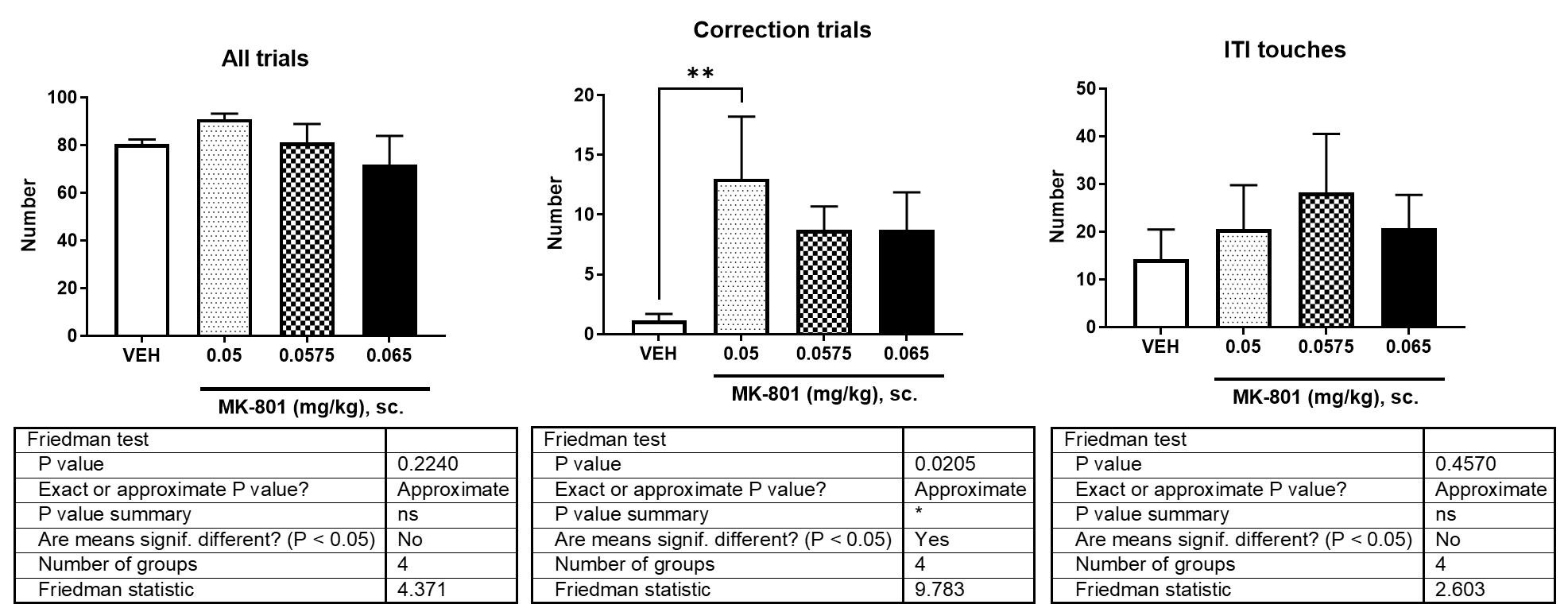
**

**Supplementary Figure S5. Surrogate measures of the MK-801 experiment.** Data represent mean ± SEM values. Trial number: the number of all trials including corrections**;** Correction trials: the number all correction trials (not including first incorrect choices)**;** ITI touches: the number of all touches to the screen (either on the left or right response window) during the intertrial interval (ITI).


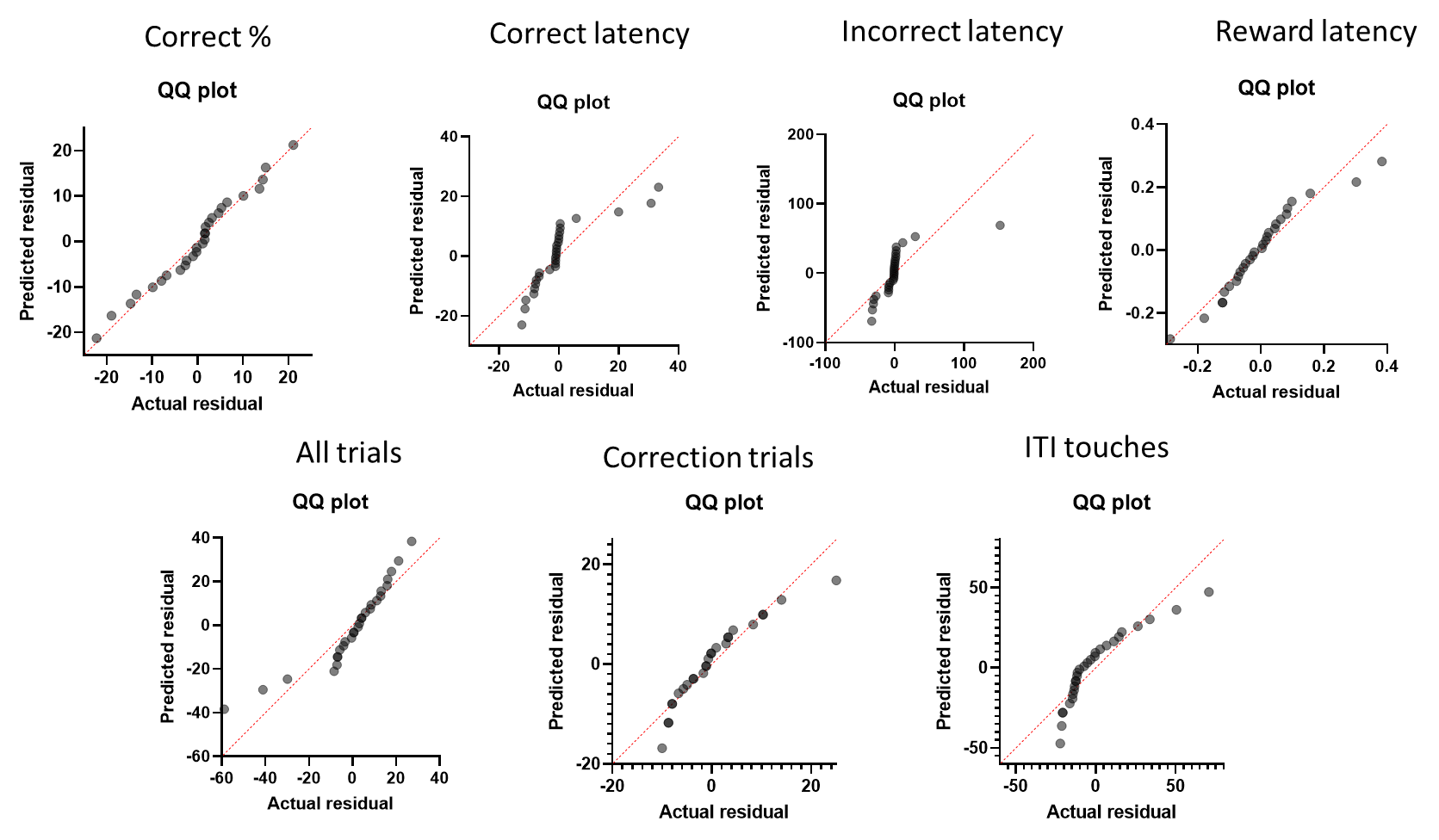


**Supplementary Figure S6. Data distribution of behavioural parameters in the MK-801 experiment**. Quantile-Quantile (QQ) plots show data deviations from normal distribution in all parameters.


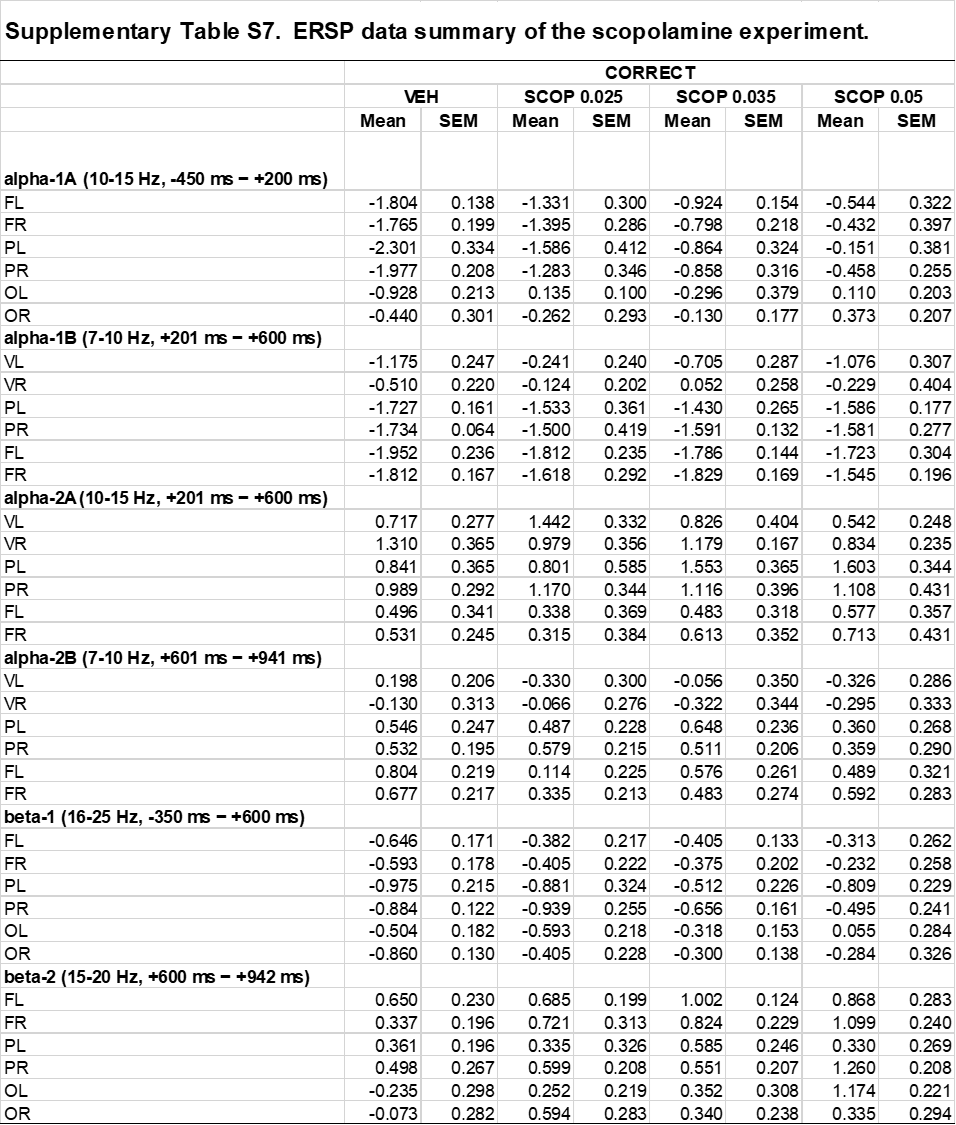


**Supplementary Table S7.** Data represents mean±SEM values of ERSP power (decibel) change relative to the baseline (-1000 − -700 ms) in the relevant time-frequency windows during correct touch responses averaged across all subjects (n=8) treated subcutaneously with vehicle (VEH) or different doses of scopolamine (0.025 mg/kg: SCOP 0.025, 0.035 mg/kg: SCOP 0.035 and 0.05 mg/kg: SCOP 0.05). Abbreviations: FL: frontal left, FR: frontal right PL: parietal left, PR: parietal right, OL: occipital left, OR: occipital right electrodes.


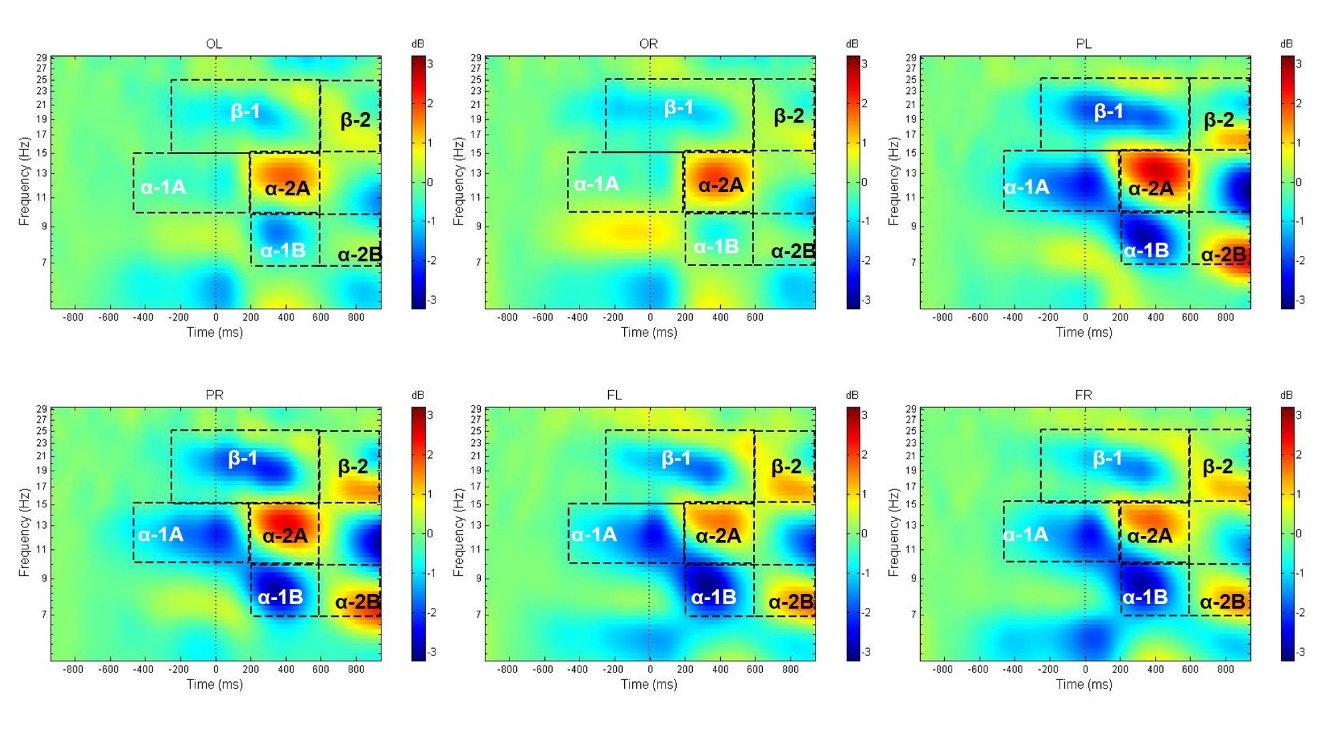


**Supplementary Figure S8.** **Group average ERSP maps of the scopolamine experiment with the relevant time-frequency windows at each electrode separately.** Plots show the mean time course of ERSP power (decibel, db) change relative to the baseline in the period from ~1000 msec before to ~1000 ms after the correct touch responses (t=0) in the frequency range of 5-30 Hz averaged across all subjects and treatment conditions in the scopolamine experiment (n=8). Black rectangles indicate the time-frequency windows of interest: alpha-1A (10-15 Hz, -450 ─ +200 ms), alpha-1B (7-10 Hz, +200 ─ +600 ms), alpha-2A (10-15 Hz, +201 ─ +600 ms) and alpha-2B (7-10 Hz, +601 ─ +942 ms), β-1 (16-25 Hz, -350 ─ +600 ms), β-2 (15-20 Hz, +600 ─ +942 ms). Abbreviations: FL: frontal left, FR: frontal right PL: parietal left, PR: parietal right, OL: occipital left, OR: occipital right electrodes.

**Supplementary Table S9.** Data represents mean±SEM values of ERSP power (decibel, db) change relative to the baseline (-1000 − -700 ms) in the relevant time-frequency windows during correct touch responses averaged across all subjects (n=7) treated subcutaneously with vehicle (VEH) or different doses of MK-801 (0.05 mg/kg: MK 0.05, 0.0565 mg/kg: MK 0.0565 and 0.065 mg/kg: MK 0.065). Abbreviations: FL: frontal left, FR: frontal right PL: parietal left, PR: parietal right, OL: occipital left, OR: occipital right electrodes.


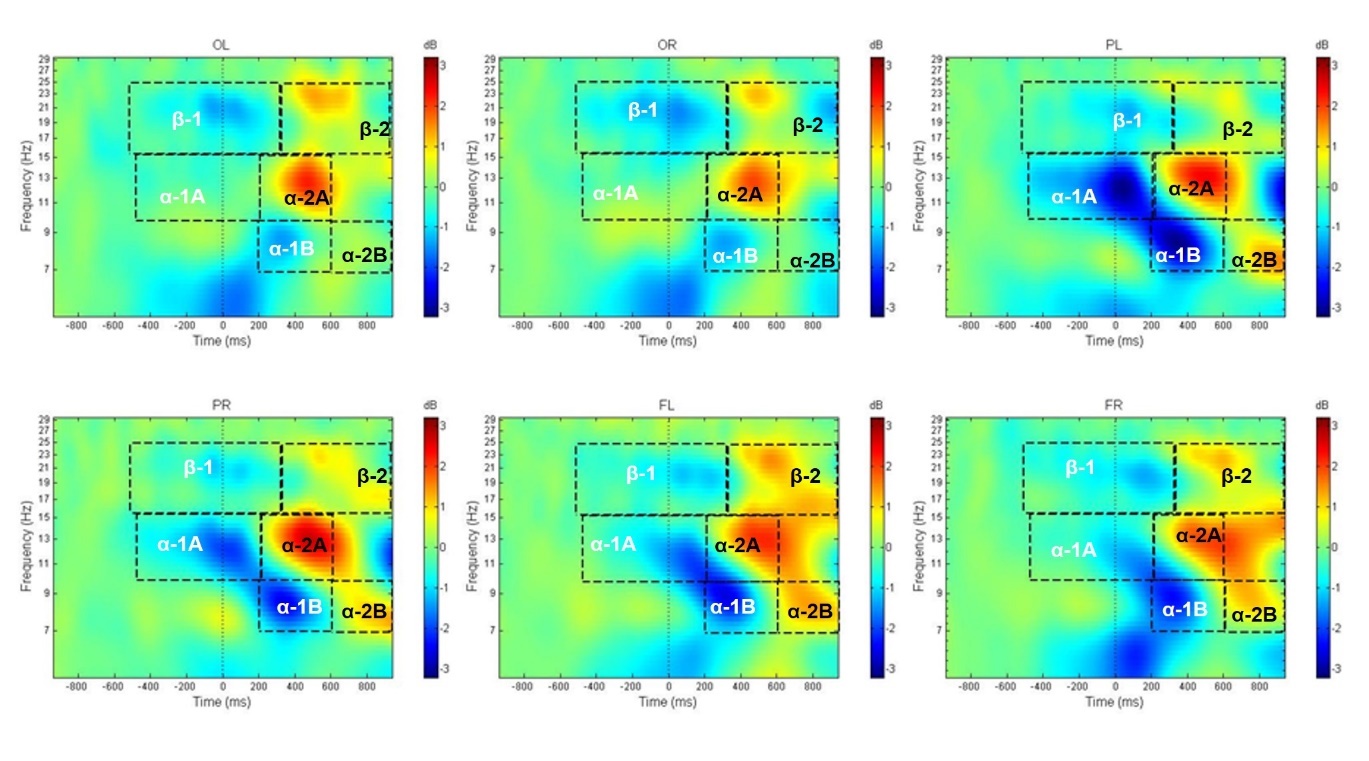


**Supplementary Figure S10.** **Group average ERSP maps of the MK-801 experiment at each electrode separately**. Plots show the mean time course of ERSP power (decibel, db) change relative to the baseline in the period from ~1000 msec before to ~1000 ms after the correct touch responses (t=0) in the frequency range of 5-30 Hz averaged across all subjects and treatment conditions in the MK-801 experiments (n=7). Black rectangles indicate the time-frequency windows of interest: alpha-1A (10-15 Hz, -450 ─ +200 ms), alpha-1B (7-10 Hz, +200 ─ +600 ms), alpha-2A (10-15 Hz, +201 ─ +600 ms) and alpha-2B (7-10 Hz, +601 ─ +942 ms), β-1 (15-25 Hz, -500 ─ +350 ms), β-2 (15-25 Hz, +350 ─ +942 ms). Abbreviations: FL: frontal left, FR: frontal right PL: parietal left, PR: parietal right, OL: occipital left, OR: occipital right electrodes.

**Supplementary Table S11.** Data represent the strength of association between correct latency time and single-trial ERSP values in each time-frequency windows in both studies, separately. Likelihood ratio test is carried out to compare the null model, i.e. model without ERSP to the model containing ERSP as fixed effect. For each model, degrees of freedom is 24.
